# Matrix Metalloproteinases Inhibitory Effects of Silver Diamine Fluoride: Influence of Time and Potassium Iodide, In Vitro

**DOI:** 10.1111/ipd.70145

**Published:** 2026-09-18

**Authors:** Lamis El Sharkasi, Lynne Bingle, Helen Rodd, Nicolas Martin, Samiya Subka, Chris Deery

**Affiliations:** ^1^ School of Dentistry Liverpool University Liverpool UK; ^2^ School of Clinical Dentistry The University of Sheffield Sheffield UK; ^3^ School of Dentistry Leeds University Leeds UK

**Keywords:** dentine caries, matrix metalloproteinases, potassium iodide, silver diamine fluoride

## Abstract

**Background:**

Silver diamine fluoride (SDF) arrests caries, partly by inhibiting matrix metalloproteinases (MMPs). Potassium iodide (KI) is often applied after SDF to mitigate tooth darkening.

**Aim:**

This in vitro study evaluated the effects of application time and potassium iodide on SDF‐mediated MMP inhibition, and assessed surface microhardness following SDF ± KI application.

**Design:**

Seventy‐two carious (ICDAS score 5 or 6) dentine blocks from 34 teeth were randomly assigned to one of four groups: baseline, SDF, SDF + KI and control. Total MMP activity was measured using a colorimetric assay. For enamel lesions, surface microhardness (VHN) was assessed on 20 enamel surfaces from 10 teeth (ICDAS 2).

**Results:**

After 1 week, SDF significantly inhibited total MMP activity by 93% (*p* < 0.001). SDF + KI showed a lower, not statistically significant level of inhibition of 55.5% (*p* = 0.07). Surface microhardness of enamel caries lesions increased by 66.8% (*p* < 0.0001) with SDF and by 71.5% (*p* < 0.0001) with SDF + KI. No significant difference was observed between 1 and 3 min of SDF application.

**Conclusion:**

In this in vitro model, SDF demonstrated strong MMP inhibition, while SDF + KI showed lower numerical inhibition, suggesting that KI may attenuate the anti‐MMP effect of SDF. SDF and SDF + KI produced similar increases in surface microhardness of non‐cavitated enamel lesions.

## Why This Paper Is Important to Pediatric Dentists

1. SDF significantly inhibits MMPs, enzymes involved in collagen degradation, which is crucial for preventing the progression of caries lesions in primary teeth.
2. Both 1‐min and 3‐min applications of SDF are equally effective in inhibiting MMPs, providing flexibility in the treatment for patients who struggle with longer application times.
3. The use of KI after SDF was associated with lower numerical MMP inhibition in this in vitro model. Paediatric dentists should be aware that the biological effects of SDF + KI may differ from SDF alone, but clinical studies are needed before conclusions can be drawn regarding effects on caries arrest.

## 1 Introduction

Dental caries is a prevalent, multifactorial disease that imposes a significant burden on affected individuals and healthcare systems globally [1, 2]. Among the minimally invasive options for managing caries, silver diamine fluoride (SDF) has emerged as a cost‐effective and clinically validated material. SDF is an alkaline solution composed of silver and fluoride ions, which exert a dual action: fluoride promotes remineralisation, while silver exhibits potent antimicrobial effects against cariogenic bacteria such as ‘ *Streptococcus mutans* ’ [3, 4].

In addition to these established actions, SDF has been shown to inhibit matrix metalloproteinases (MMPs), particularly MMP‐2, MMP‐8 and MMP‐9, enzymes that play a central role in the degradation of collagen within demineralised dentine [5, 6]. The silver ions in SDF can chelate with zinc and calcium at the catalytic domains of MMPs, suppressing their proteolytic activity [5, 7]. These MMPs are secreted by odontoblasts and immune cells and remain latent in mineralised dentine until activated by acidic conditions, such as those present in caries lesions [8]. Therefore, inhibition of MMPs contributes significantly to the caries‐arresting potential of SDF by stabilising the organic dentinal matrix [4, 9, 10].

To address the cosmetic concern of black staining caused by SDF, potassium iodide (KI) is sometimes applied immediately after SDF application. KI reacts with free silver ions to form silver iodide (AgI), a pale‐yellow precipitate [8]. However, this reaction could potentially reduce the bioavailability of silver ions and interfere with their antibacterial and MMP‐inhibitory actions. Recent studies suggest KI may attenuate the therapeutic effects of SDF, although results remain inconsistent [3, 7, 11, 12, 13, 14, 15].

Despite the well‐documented clinical efficacy of SDF in arresting dental caries, two important considerations remain insufficiently investigated. The first concerns the use of potassium iodide (KI) following SDF application, which is intended to mitigate the characteristic black discolouration but may also influence matrix metalloproteinase (MMP) activity [7, 16]. Although KI effectively reduces staining, its use may reduce the effectiveness of SDF [15].

Secondly, although current recommendations suggest an SDF application time of 1 to 3 min [7], achieving even a 1‐min application period can be challenging in young or uncooperative patients, highlighting the need to determine the minimum effective duration for optimal therapeutic benefit. The present in vitro study aims to: (1) evaluate the medium‐term inhibitory effects of SDF on matrix metalloproteinase (MMP) activity and dentinal collagen degradation, with and without the use of potassium iodide (KI), across different application times on natural carious dentine; and (2) assess the effect of SDF, again with and without KI, on the surface microhardness of non‐cavitated natural caries lesions.

## 2 Materials and Methods

### 2.1 Sample Preparation

Extracted human primary molar teeth with varying caries severity were retrieved from a biobank (stored at −80°C), where collagen structure and enzyme activity remain minimally affected due to slowed biochemical processes [16]. Figure 1 illustrates the overall methods, grouping and sample size in each group. Teeth were thawed overnight at 4°C before use. Prior to extraction, teeth had been diagnosed by a trained and calibrated ICDAS examiner as part of a previous study [17]. Ethical approval was obtained from National Health Service Research Ethics Committee (NHS REC; Reference 12/YH/0214) and from Sheffield Teaching Hospitals Research Governance Department (Protocol Number STH16301), and all parents provided written informed consent with child assent for tooth retention and use.

**FIGURE 1:**
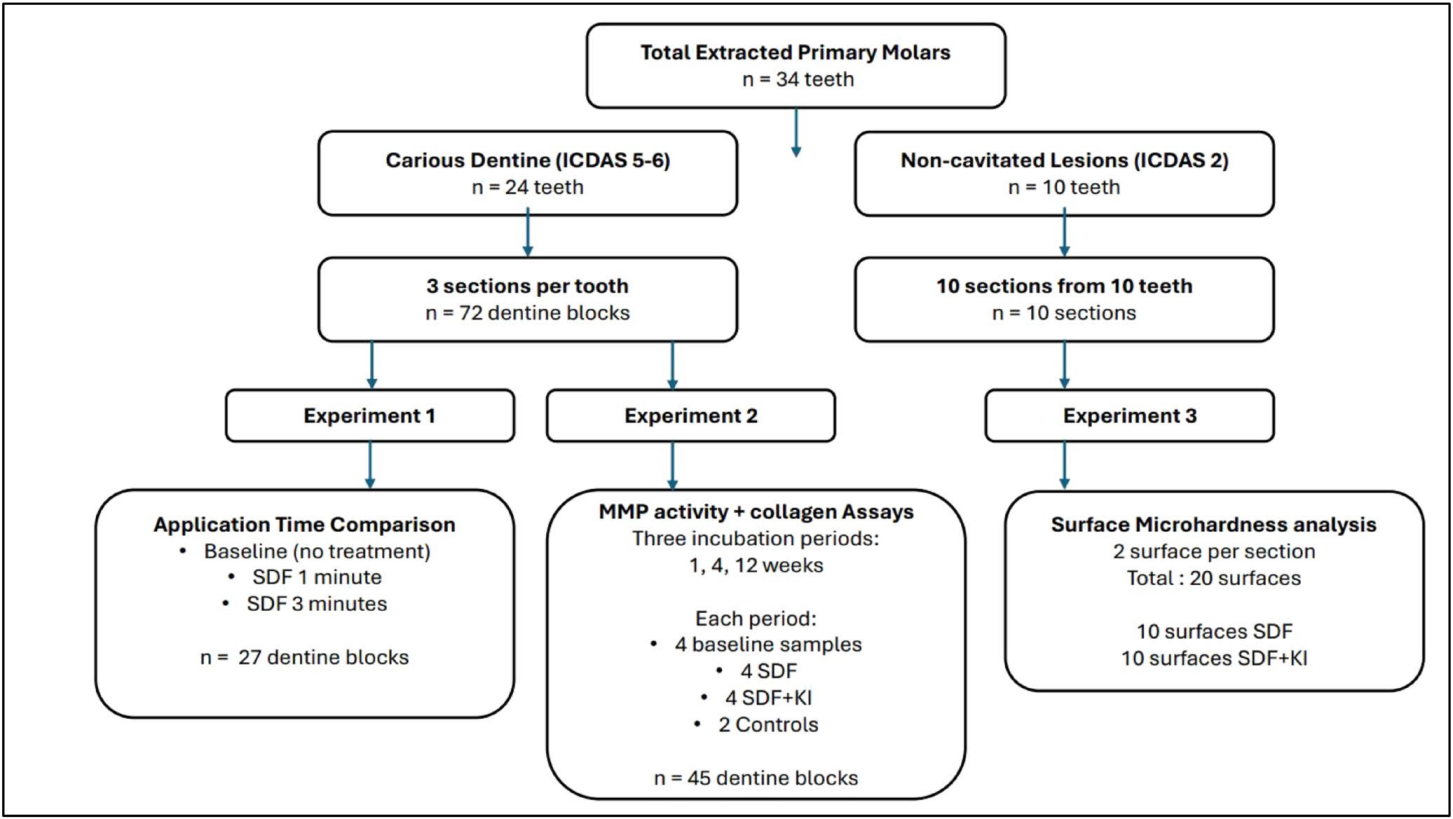
Distribution of extracted primary teeth (*n* = 34) across experimental groups.

In total, 34 extracted teeth were used. Twenty‐four teeth with cavitation (ICDAS 5–6), which permits access to carious dentine, were examined to assess the inhibitory effect of 38% SDF with and without KI (Riva Star, SDI, Bayswater Vic, Australia) on MMPs and collagen degradation using MMP activity and hydroxyproline assays. Ten teeth with non‐cavitated lesions (ICDAS 2) were used to evaluate the effect of SDF ± KI on surface microhardness using the Vickers hardness test.

Teeth were sectioned mesio‐distally into 500 μm thick slices [17]. For MMP activity assays, three sections per tooth (24 teeth × 3 slices each tooth) yielded 72 carious dentine blocks (3 mm × 3 mm × 500 μm). For microhardness testing, 10 sections from 10 primary molars (ICDAS 2) provided 20 surfaces (two per section). Enamel surfaces were polished using P1500 grit silicon carbide paper (Struers Ltd., Rotherham, UK) under water coolant for 30 s before SDF application to standardise surface texture. Surface roughness was not quantified.

### 2.2 Saliva Collection and Preparation

Ethical approval for saliva collection was granted by the University of Sheffield Ethics Committee (Reference Number 040965 02/08/2021). To mimic the oral environment, carious dentine blocks were incubated in natural saliva for SDF experiments and collagen degradation measurements. Unstimulated saliva (10 mL) was collected from a single donor (a 39‐year‐old female with clear medical and medication history) on two occasions at the same time of day. The donor refrained from eating or drinking for 1 h prior to collection and rinsed her mouth with water before sampling. (Note: These experiments were conducted during the COVID‐19 pandemic; using saliva from the researcher reduced cross‐infection risk.) Saliva was centrifuged at 2000 rpm for 10 min to remove debris and high molecular weight proteins (GenFuge, Proyen, UK), then sterilised using a 0.22 μm syringe filter (StarLab International GmbH, Hamburg, Germany) to eliminate bacteria. Aliquots of 400 μL were stored in glass vials.

### 2.3 Analysis of Residual MMP Activity Using Generic MMP Assay

The SensoLyte Generic MMP Assay kit (Anaspec, San Jose, CA, USA) was used to measure residual MMP activity in carious teeth under two experimental conditions: Experiment 1 examined carious lesions before and after 1‐ or 3‐min SDF application; Experiment 2 examined carious lesions before and after SDF ± KI application following three incubation periods in natural saliva (1, 4 and 12 weeks).

***Experiment 1:*** Three serial carious dentine blocks from each tooth were used. Baseline MMP activity was measured from the first block. The second and third blocks were treated with 38% SDF for 1 and 3 min, respectively. Control samples received no treatment. Following SDF treatment, blocks were dipped into wells containing 250 μL distilled water (dH_2_O) for 2 min to remove absorbed SDF [18]. Residual MMP activity was then measured.

***Experiment 2:*** Three serial carious dentine blocks from each tooth were used. Baseline enzyme activity was measured from the first block. The second block was treated with 38% SDF for 1 min. The third block received SDF for 1 min followed by KI application until white precipitation ceased. Blocks were then dipped into wells containing 250 μL dH_2_O for 2 min to remove absorbed SDF [19]. Samples were incubated in natural saliva and divided into three groups by incubation period. Each group contained four baseline samples, four SDF‐treated samples, four SDF + KI‐treated samples and two untreated controls. Following each incubation period, residual MMP activity was measured.

***Assay Procedure:*** Chromogenic thiopeptide substrate solution was prepared by adding 100 μL MMP substrate to 4.9 μL assay buffer (1:50 dilution). Samples were incubated in substrate solution for 90 min at 37°C, then removed. Substrate degraded by MMPs was measured at OD 412 nm using a spectrophotometer (Tecan, Männedorf, Switzerland).

### 2.4 Analysis of Collagen Integrity Using Hydroxyproline Assay

A hydroxyproline colorimetric assay kit (ab222941, Abcam, Cambridge, UK) was used to measure collagen degradation in the incubation media (natural saliva) at different time points following SDF ± KI application. Incubation medium (100 μL) was collected, transferred to polypropylene vials containing 100 μL of 10 N NaOH (Sigma Aldrich, Poole, UK), hydrolysed at 120°C for 1 h and cooled. Residual NaOH was neutralised by adding 100 μL of 10 N HCl (Sigma Aldrich, UK). Vials were centrifuged (GenFuge, Progen, UK) at 10 000× *g* for 5 min to pellet insoluble debris, and supernatants were transferred to fresh vials. Aliquots (10 μL) from each vial and standard dilutions were transferred to a 96‐well plate and evaporated at 65°C on a microplate incubator (JENCONS PLS, Bedfordshire, UK) until crystalline residue formed. Oxidation solution (100 μL), prepared by mixing 6 μL chloramine T concentrate with 94 μL oxidation buffer, was added to each well and incubated at room temperature for 20 min. Developer (50 μL) was added and incubated for 5 min at 37°C, followed by 50 μL DMAB concentrate. The sealed plate was incubated at 65°C for 45 min, and absorbance was measured at OD 560 nm using a spectrophotometer.

### 2.5 Analysis of Surface Microhardness

***Experiment 3:*** Microhardness was measured on enamel surfaces (ICDAS 2) before and after SDF ± KI application. Ten surfaces were used for each comparison. Baseline microhardness was measured using a Vickers microhardness tester (Foundrax, UK) with a 300 g load and 11 s dwell time [20]. Six measurements were taken at least 100 μm apart, and the mean was calculated for each sample. SDF was applied to the enamel surface for 1 min, and samples were immersed in wells containing 250 μL deionised water until re‐measurement [18]. Final microhardness was measured alongside the baseline indentation (six measurements) and recorded as the final Vickers hardness number (VHN). For SDF + KI comparison, the same procedure was followed.

### 2.6 Statistical Analysis

Data distribution normality was verified using the Shapiro–Wilk test. Paired *t*‐tests compared different application times (1 vs. 3 min) within the same treatment group. One‐way ANOVA with Tukey's post hoc test compared treatment types (SDF vs. SDF + KI) across multiple incubation periods (1, 4 and 12 weeks). Statistical significance was set at *p* < 0.05. All analyses were performed using GraphPad Prism version 8 (GraphPad Software, San Diego, CA, USA). Although three independent variables were involved (treatment type, application time and incubation period), these were analysed in separate focused experiments due to sample size constraints and experimental clarity.

## 3 Results

### 3.1 Percentage of Inhibition of Total MMP Activity in Caries Lesions After Different Application Times of SDF

The residual MMP activity at the two application times of SDF and the control were measured and indicated that SDF treatment for both 1 and 3 min resulted in significant MMP inhibition by 94% and 97%, respectively, compared to baseline and control (*p* < 0.001). The difference in MMP inhibition between the two application times was not statistically significant (*p* = 0.0982) (Figure 2).

**FIGURE 2:**
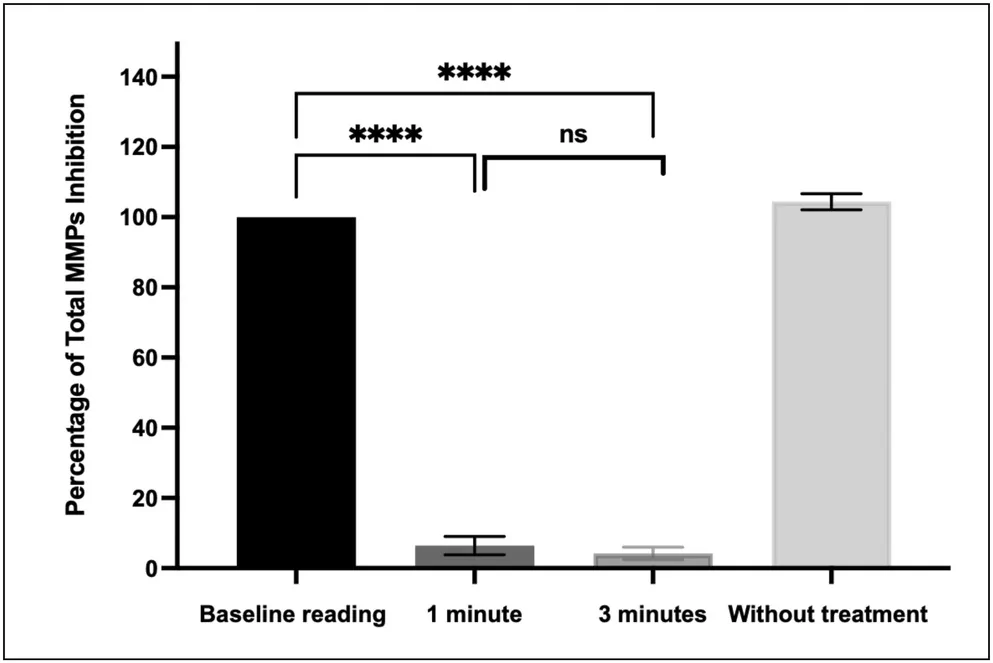
Bar graph showing total MMP residual activity in caries lesions after two SDF application times (1, 3 min). Data were analysed using ANOVA and the Tukey multiple comparison test (****p‐value < 0.0001).

### 3.2 Percentage of Inhibition of Total MMP Activity in Caries Lesions After SDF and SDF + KI Application

After 1 week of incubation in saliva, residual MMP activity was statistically significantly lower in the SDF‐treated group compared to baseline (*p* < 0.001) with inhibition by 93%. MMP activity was also statistically significantly reduced in the SDF‐treated group compared to the control group (*p* < 0.001). Residual MMP activity in the SDF‐treated group was also significantly lower than that in the SDF + KI‐treated group (*p* = 0.02); however, the difference in inhibition between baseline and SDF + KI (inhibition by 55.5%) was not statistically significant (*p* = 0.07) (Figure 3a).

**FIGURE 3:**
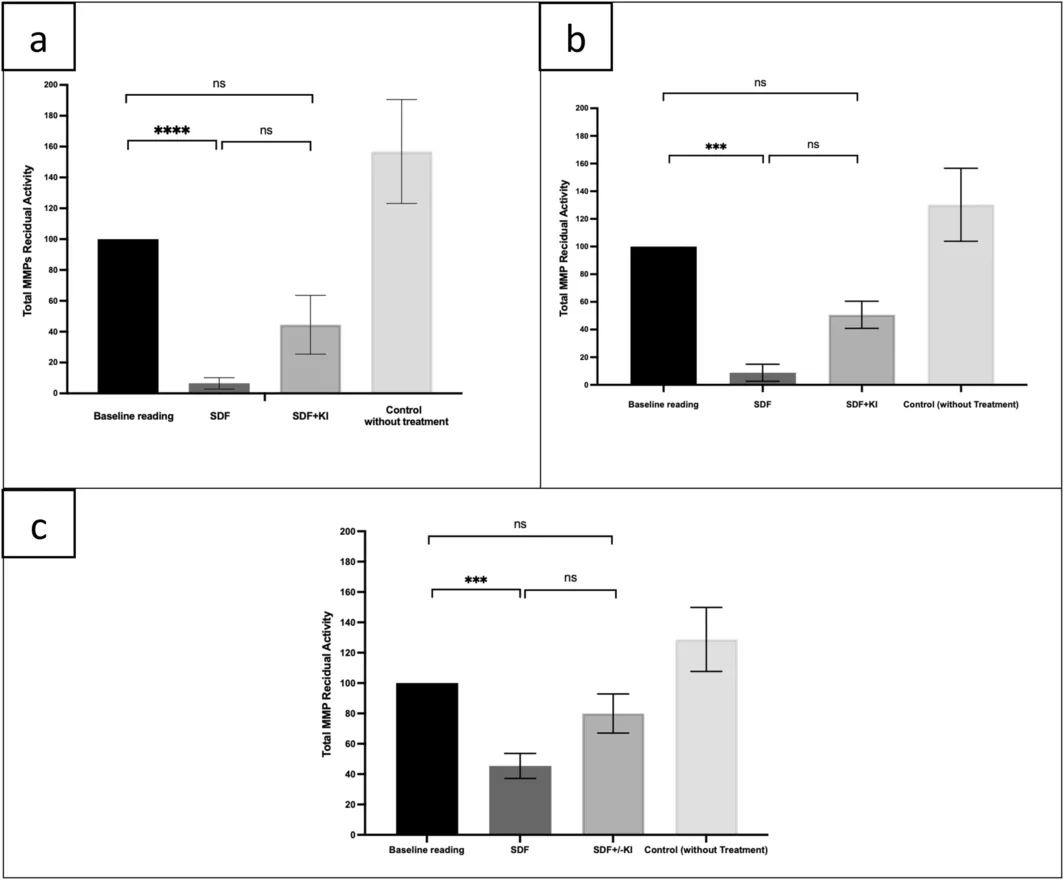
Bar graphs showing total MMP residual activity in caries lesions after application of SDF and SDF + KI measured at three incubation periods in natural saliva (a) 1 week, (b) 4 weeks, (c) 12 weeks. Data were analysed using ANOVA and the Tukey multiple comparison test (*p‐value < 0.05, **p‐value < 0.01, ***p‐value < 0.001, ****p‐value < 0.0001).

After 4 weeks of incubation, residual MMP activity was measured and found to be significantly lower in the SDF‐treated group than baseline and control (*p* < 0.001) as SDF inhibited total MMP activity by 91% (*p* < 0.001), while the application of SDF + KI decreased activity by 48%. The difference in residual MMP activity between the baseline group and the SDF + KI treated group was not statistically significant (*p* = 0.06) (Figure 3b).

After 12 weeks of incubation, SDF significantly inhibited total MMP activity by 55%, while application of SDF + KI reduced activity by 20%. The difference between endpoint value of MMP activity at baseline was significantly lower after SDF treatment (*p* = 0.002), while the decrease in activity in the SDF + KI group was not statistically significant (*p* = 0.31) (Figure 3c).

SDF showed a significant longer‐term inhibitory effect (up to 12 weeks) on MMP activity compared to SDF + KI. Within the same experimental treatments, the decrease in MMP activity did not change significantly between week one and week 4 (*p* = 0.43). However, the decrease between week one and week 12 and between week 4 and week 12 was statistically significant (*p* < 0.001). In the SDF + KI treatment group, there was no statistically significant decrease in MMP activity between all incubation times (*p* = 0.89).

### 3.3 Quantitative Measurement of the Extent of Collagen Degradation in Caries Lesions After SDF and SDF + KI Application

After 1 week of incubation, mean hydroxyproline concentration (HYP) was significantly lower (*p* = 0.001) in SDF‐treated group (0.141 ± 0.024) than in control group (0.402 ± 0.074). In SDF + KI treated group, the mean HYP concentration (0.245 ± 0.053) was significantly higher than in SDF‐treated group (*p* = 0.041) (Figure 4a).

**FIGURE 4:**
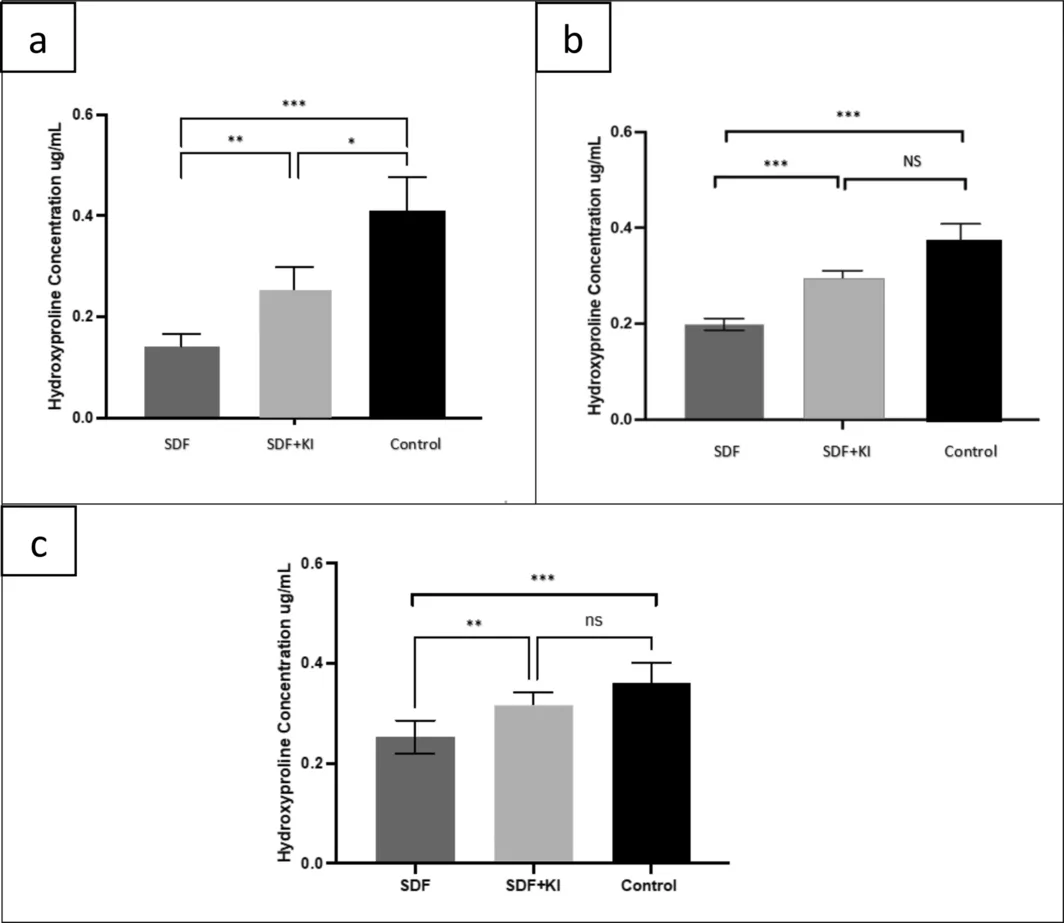
HYP assay showing the quantity of denatured collagen in caries lesions following SDF and SDF + KI application measured after (a) 1 week, (b) 4 weeks and (c) 12 weeks of incubation in natural saliva. Data were analysed using ANOVA followed by Tukey multiple comparison test (*p‐value < 0.05, **p‐value < 0.01, ***p‐value < 0.001).

A similar trend was observed after 4 weeks as the SDF‐treated group showed a statistically significant decrease (*p* < 0.001) in the concentration of HYP compared to the control group (0.198 ± 0.012 and 0.373 ± 0.035, respectively) and the SDF + KI group had a statistically significant (*p* < 0.001) higher concentration of HYP (0.295 ± 0.015) than the SDF‐treated group (Figure 4b).

After 12 weeks of incubation, mean HYP concentration was significantly lower in the SDF‐treated group (0.252 ± 0.033) than in the control group (0.359 ± 0.042) (*p* = 0.009) and SDF + KI‐treated group (0.324 ± 0.018) (*p* = 0.025). The difference between the SDF + KI treated group and control group was not statistically significant (*p* = 0.397) (Figure 4c).

### 3.4 Effect of SDF ± KI Application on the Microhardness of Non‐Cavitated Caries Lesions

In SDF‐treated group (*n* = 10), there was a statistically significant difference (*p* < 0.0001) in VHN between baseline (201.8 ± 26) and SDF treatment (336.6 ± 48). The mean percentage increase in microhardness after treatment was 66.8%. For SDF + KI treatment group (*n* = 10), there was also a statistically significant difference (*p* < 0.0001) in VHN between baseline (189 ± 30) and SDF + KI treatment (324.2 ± 25) where the mean percentage increase in microhardness after treatment was 71.5%. The difference in microhardness between SDF treatment and SDF + KI was not statistically significant (*p* = 0.480) (Figure 5).

**FIGURE 5:**
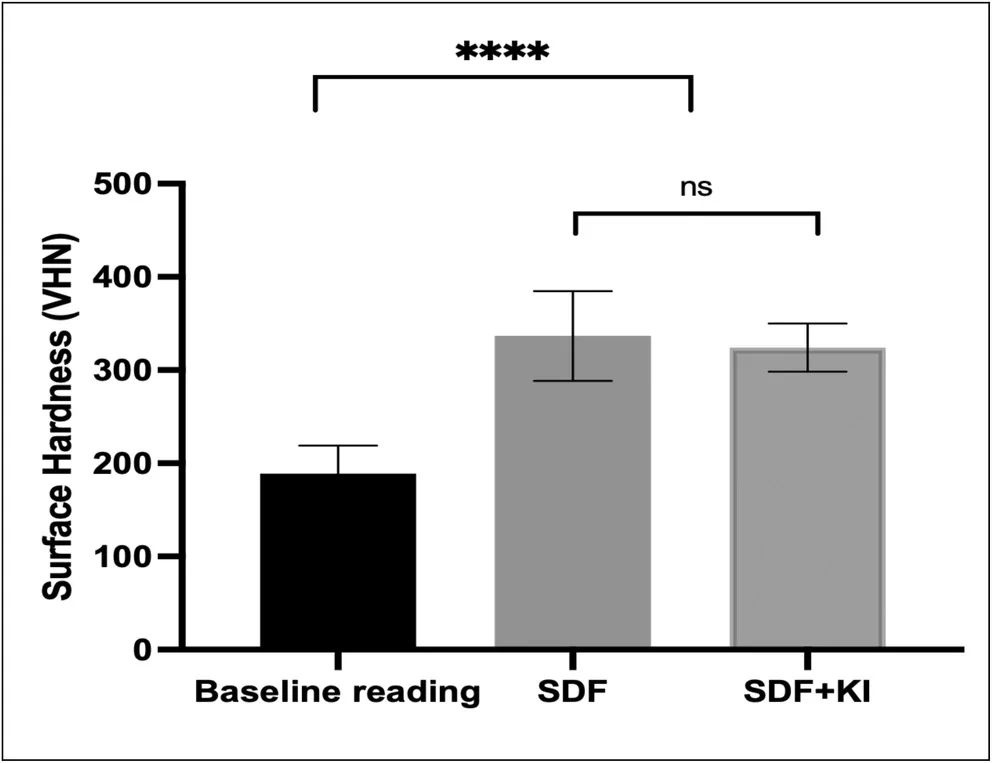
Bar graph showing Vickers microhardness number of enamel surfaces with ICDAS at baseline and following SDF and SDF + KI (****p‐value < 0.0001).

## 4 Discussion

Superficial sound dentine compared to that nearer the pulp is more densely packed with better organised collagen fibrils, with smaller and fewer tubules, leading to a higher mineral content, making the organic matrix more resistant to early enzymatic cleavage. Generally, the dentine closer to the pulp has greater MMP activity, specifically MMP‐2 and MMP‐9 [21]. Although, all types of MMP are present throughout the dentine. Levels of MMP increase with the presence and severity of caries [22].

Turton et al.'s in vivo study demonstrated that the application of KI results in less caries arrest [15]. The present in vitro study confirms this finding using the objective measure of surface hardness. It also presents a mechanism for this finding, that is, KI negatively impacts the caries inhibitory potential, specifically affecting MMP activity and collagen degradation.

Percentage of inhibition of total MMP activity in caries lesions after different application times of SDF. Existing recommendations suggest an application time for SDF of between 1 and 3 min to allow adequate adsorption within the lesion [8, 23]. A 3 min or even a 1 min application poses a significant challenge for some patients. Laboratory studies have primarily focused on the impact of application duration on remineralisation which suggests that both 30 s [24] and 1 min [23] application times provide comparable results to a 3 min application. Studies have used the precipitation of silver chloride and calcium phosphate as measures demonstrating remineralisation in dentine [24] and enamel [23]. Results from the present study show in addition robust MMP inhibition by SDF, independent of the application time. A review of SDF application protocols has reported variation, with application times ranging from 10 to 240 s. There is a need to identify the optimal application time [25].

Percentage of inhibition of total MMP activity in caries lesions after SDF and SDF + KI application. Inhibition of MMPs is important to prevent lesion progression. This study emphasises SDF efficacy as an inhibitor of total MMP activity in caries lesions, with a remarkable inhibition rate of 93% after 1 week. These results are in agreement with the study by Mei et al., which demonstrated significant inhibition of MMP2, MMP8 and MMP9 by 79%, 94% and 82%, respectively [5]. It is important to note that the study by Mei et al. used recombinant MMPs, which may have overlooked the nuanced complexity of matrix‐bound MMPs in natural carious dentine [4].

Concerns about discolouration of caries lesions following SDF treatment have led to the use of KI to mitigate this side effect [8]. While several studies have validated the effectiveness of KI in reducing discolouration, concerns have been raised about the impact on the effectiveness of SDF [9, 10, 11, 26]. Several studies have investigated this combination and its effects on antibacterial properties and bond strength, with conflicting results [12, 13]. The current study is the first to investigate the influence of KI on the anti‐MMP potential of SDF on natural carious lesions and shows a significant reduction in the anti‐MMP effect of SDF following the application of KI, from 93% to 55.5%. This finding supports the impairment of the caries inhibitory effect of SDF when KI is applied. The mechanism of this negative effect of KI requires further investigation.

The findings suggest that the addition of KI may reduce the anti‐MMP activity of SDF in this in vitro model. This may be relevant when considering the balance between reducing discolouration and preserving the biological effects of SDF. However, as this study evaluated laboratory surrogate outcomes rather than clinical caries arrest, the findings should be interpreted cautiously and require confirmation in clinical studies.

Quantitative measurement of the extent of collagen degradation in caries lesions after SDF and SDF + KI application. To measure the degree of collagen degradation after SDF treatment, carious dentine blocks were incubated in natural saliva. As collagen degradation products seep into an incubation medium, the assessment of the HYP concentration in natural saliva allowed direct assessment of collagen degradation and the effect of SDF in preventing this degradation. This assay is based on the assumption that 90% of the dry mass of dentine contains Type I collagen and 10% of its mass consists of HYP [27, 28]. Our results showed that SDF is a potent inhibitor of collagen degradation in dentine caries lesions. The levels of HYP were significantly lower in the first week after SDF treatment when compared to the control. This is supported by the work of Mei et al., who investigated the inhibitory effect of different concentrations of SDF on collagen degradation in sound dentine and found that the concentration of HYP was significantly lower in the SDF group compared to the control group [4].

Effect of SDF ± KI application on the microhardness of non‐cavitated caries lesions. The present study found that the application of SDF resulted in a significant 66.8% increase in microhardness of non‐cavitated caries lesions. A smaller percentage increase in microhardness (32%) was reported in the study by Punhagui et al. [23]. However, they used induced enamel lesions, resulting in a much lower baseline hardness than baseline measurements on natural enamel lesions used in the present study. These findings are due to the remineralising effect of fluoride, rather than SDF's inhibition of MMP's, which is confined to dentinal caries. In vivo, the antibacterial properties of silver are likely to provide a further benefit. Kim et al. found that 38% SDF had a more substantial remineralising effect on artificially induced enamel lesions than 5% sodium fluoride [29]. In the present study, the application of KI after SDF did not affect the ability of SDF to increase the surface microhardness of enamel lesions. These results indicate that SDF could be considered as the first choice fluoride remineralising agent for initial enamel caries and that KI can be used without detriment to minimise aesthetic concerns.

Strength and limitations. By using a convenience sample and the use of human teeth with naturally occurring caries lesions allowed us to investigate MMP activity in or near their natural environment in caries lesions. This was essential for accurate analysis and distinguishes our study from previous investigations in which demineralised healthy dentine beams or recombinant protein have been used. A limitation of our study is that the change in microhardness was measured over a relatively short time. Measuring the long‐term effects of SDF ± KI in remineralising enamel lesions with longer incubation times could have provided helpful information to predict the longevity of these results. Another limitation is the use of different sections of the carious lesion; however, preliminary experiments confirmed the suitability of this method by showing nearly identical MMP activity measurements in sections of the same carious lesion.

This study has some limitations. First, the natural saliva used as the incubation medium was not renewed throughout the experimental periods. This may have led to reduced enzymatic activity over time due to potential degradation, inhibition, pH shifts and accumulation of byproducts, which do not fully replicate the dynamic oral environment. Second, the samples were not subjected to demineralisation–remineralisation cycles, and therefore the model did not simulate the natural pH fluctuations that occur in the oral cavity. These cycles can influence collagen exposure and MMP activation. While both limitations may affect the extent to which the findings reflect in vivo conditions, the controlled and consistent setup across all groups allows for valid comparative analysis of MMP activity and collagen degradation over time. Future studies should consider medium renewal and pH cycling to better mimic the clinical oral environment.

This investigation has conclusions of clinical relevance:

1. The caries‐inhibiting effects of SDF include short‐ and medium‐term inhibition of total MMP activity and effective prevention of collagen matrix degradation in dentine caries.
2. SDF increases the surface microhardness of the enamel surface in non‐cavitated caries lesions.
3. There were no significant differences between 1 and 3 min application times of SDF in terms of MMP inhibition.
4. SDF demonstrated marked inhibition of total MMP activity and reduced collagen degradation in carious primary dentine. The addition of KI was associated with lower numerical MMP inhibition, suggesting that KI may attenuate this biological effect. No difference was observed between 1‐ and 3‐min SDF application times, and SDF + KI did not significantly alter the surface microhardness response of non‐cavitated enamel lesions compared with SDF alone. Further clinical research is needed to determine whether the observed laboratory differences translate into differences in caries arrest outcomes.

## Author Contributions

L.E.S. contributed to the conception, design, data acquisition and interpretation, drafted and critically revised the manuscript. L.B. contributed to the conception, design, data acquisition and interpretation, drafted and critically revised the manuscript. H.R. contributed to the conception, the design, data acquisition and interpretation, drafted and critically revised the manuscript. N.M. contributed to the conception, design, data acquisition and interpretation, drafted and critically revised the manuscript. S.S. contributed to the conception, design, data acquisition and interpretation, drafted and critically revised the manuscript. C.D. contributed to the conception, design, data acquisition and interpretation, drafted and critically revised the manuscript. All authors gave their final approval and agreed to be accountable for all aspects of the work.

## Funding

Dr. Lamis El Sharkasi and this research was funded by a scholarship from the Libyan Government.

## Ethics Statement

Ethical approval for the use of these teeth was obtained from the National Health Services Research Ethics Committee (NHS REC; Reference 12/YH/0214), Sheffield Teaching Hospitals Research Governance Department (Protocol Number STH16301) and ethical approval for collecting normal human saliva was granted by the University of Sheffield Ethics Committee (Reference Number 040965; 02/08/2021).

## Consent

All parents/carers gave informed written consent for the use of their child's extracted tooth.

## Conflicts of Interest

The authors declare no conflicts of interest.

## Data Availability

The raw data is available from the authors on request.
